# Mitochondrial Function in Permeabilized Cardiomyocytes Is Largely Preserved in the Senescent Rat Myocardium

**DOI:** 10.1371/journal.pone.0043003

**Published:** 2012-08-09

**Authors:** Martin Picard, Kathryn J. Wright, Darmyn Ritchie, Melissa M. Thomas, Russell T. Hepple

**Affiliations:** 1 Department of Kinesiology, Department of Medicine, McGill University, Montreal, Quebec, Canada; 2 Muscle & Aging Laboratory, Faculty of Kinesiology, University of Calgary, Calgary, Alberta, Canada; Université Joseph Fourier, France

## Abstract

The aging heart is characterized by a progressive decline in contractile function and diastolic relaxation. Amongst the factors implicated in these changes is a progressive replacement fibrosis secondary to cardiomyoctye death, oxidative damage, and energetic deficit, each of which may be secondary to impaired mitochondrial function. Here, we performed an in-depth examination of mitochondrial function in saponin-permeabilized cardiomyocyte bundles, a preparation where all mitochondria are represented and their structure intact, from young adult (YA) and senescent (SEN) rats (n = 8 per group). When accounting for increased fibrosis (+19%, P<0.01) and proportional decrease in citrate synthase activity in the SEN myocardium (−23%, P<0.05), mitochondrial respiration and reactive oxygen species (H_2_O_2_) emission across a range of energized states was similar between age groups. Accordingly, the abundance of electron transport chain proteins was also unchanged. Likewise, except for CuZnSOD (−37%, P<0.05), the activity of antioxidant enzymes was unaltered with aging. Although time to mitochondrial permeability transition pore (mPTP) opening was decreased (−25%, P<0.05) in the SEN heart, suggesting sensitization to apoptotic stimuli, this was not associated with a difference in apoptotic index measured by ELISA. Collectively, our results suggest that the function of existing cardiac ventricular mitochondria is relatively preserved in SEN rat heart when measured in permeabilized cells.

## Introduction

Mitochondrial dysfunction has been postulated to play a major role in the process leading to cellular senescence and to aging in general [1], [2], [3]. In the heart, major age-related functional impairments of the myocardium include i) increased stiffness – due both to fibrosis [4], [5], [6] and impaired diastolic relaxation [7], ii) decreased systolic pressure development and fractional shortening [7], and iii) compensatory left ventricular hypertrophy [8], [9], [10] in response to apoptotic loss of cardiomyocytes [5], [11], [12], [13]. In general, left ventricular hypertrophy in humans is associated with poor prognosis including heart failure, exercise intolerance and early death [14], [15]. It is thus important to identify the factors that cause pathological enlargement and contractile dysfunction of the aged heart.

These changes in heart structure and function from young adulthood (YA) to senescence (SEN) could be explained by changes in three main aspects of mitochondrial function. First, increased vulnerability to the pro-apoptotic event of mitochondrial permeability transition pore (mPTP) opening with age [16], [17], [18] may cause loss of cardiomyocytes by apoptotic cell death [5], [19], contributing to weakening of the myocardium and replacement of cardiomyocytes with fibrotic tissue [4], [5], [7]. Second, increased reactive oxygen species (ROS) production by mitochondria [17] may contribute to impaired contractility and apoptotic signaling and could contribute to loss of mitochondrial respiratory capacity due to damage to mitochondrial DNA and other molecules. Finally, it has been suggested that oxidative capacity and ATP production within cardiomyocytes decreases, either due to a loss of mitochondrial content [19], [20] and/or to an intrinsic loss of mitochondrial respiratory capacity [11], [17]. Failure to maintain sufficiently high ATP levels within cardiomyocytes could result in impaired contraction [7] as well as impaired relaxation secondary to suboptimal removal of Ca^2+^ from cytoplasm by sarcoplasmic reticulum and mitochondria [4], [5], [6], [21]. Thus, because age-related decay of specific mitochondrial functions could contribute to aging of the heart and favor the development of heart failure, there is a need to establish whether mitochondrial function is altered in the aged heart.

Most previous studies have assessed different aspects of mitochondrial function using isolated mitochondria. In this study, we sought to re-evaluate the hypothesis that intrinsic mitochondrial function in heart is impaired with aging using an extensive assessment of mitochondrial function in permeabilized cells where mitochondrial morphology is preserved [22], [23]. We measured mitochondrial function using permeabilized cardiomyocytes prepared from the myocardium of young adult (YA; 8 mo old) and senescent (SEN; 36 mo old) Fisher 344/Brown Norway F1 hybrid (F344BN) rats, as well as histological assessment of fibrosis and biochemical measurements for markers of mitochondrial content, abundance of electron transport chain proteins, apoptosis and antioxidant capacity. Overall, we find that mitochondria from senescent hearts have relatively preserved mitochondrial function, and that the observed differences with aging are in several respects more modest than what has been previously suggested by studies on isolated organelles.

**Table 1:** Animal and heart weights.

	YA	SEN	SEN/YA ratio
**Heart mass (mg)**	852±12	1,278±174 **	1.50
**Body mass (g)**	400±21	539±30 **	1.35
**Heart/body mass (%)**	0.214±0.013	0.234±0.011 *	1.09

YA  =  young adult, SEN  =  senescent. Values are means ± S.E.M.; **P<0.001 vs YA, *P<0.01 vs YA, n = 7–8 per age group.

## Materials and Methods

### Animals and surgical methods

All experimental procedures were approved by the Animal Care Committee at the University of Calgary (protocol ID BI09R-11). Male Fischer 344× Brown Norway F1-hybrid (F344BN) rats were obtained from the colony maintained by the National Institute on Aging (USA) at 8–10 months (young adult – YA) or 35–36 months (Senescent – SEN). At this age, SEN rats exhibit significant age-related cardiac dysfunction and hypertrophy [7]. Upon arrival, animals were kept for about 48 hours in a 12:12 h light-dark cycle, at an ambient temperature 23°C, and provided food and water *ad libitum*. On the day of experiments, animals were anesthetized with 55–65 mg × kg^−1^ Sodium Pentobarbital (I.P.). For animals used in mitochondrial function assays (n = 8 per age group), removal of the beating heart was performed immediately following resection of hind-limb skeletal muscles (for another study). Upon removal, the heart was immediately put in ice-cold relaxing Buffer A, quickly blotted, and weighed. The apex was then removed and the left ventricle dissected for preparation of permeabilized myofibers (one half) and for biochemical experiments (one half). Full details of experimental procedures, buffers and reagents for mitochondrial functional assays are provided in [24], and are summarized below. For animals used in *in situ* ventricular fibrosis analysis (n = 6 per age group), the beating heart was removed following hindlimb perfusion procedures reported previously [25]. Hearts were blotted dry, weighed, and subsequently a cross-section (approximately 6–8 mm thick) through both ventricles was mounted in transverse orientation on cork in optimal cutting temperature (OCT) compound before being frozen in liquid nitrogen-cooled isopentane and stored at −80°C.

**Figure 1:**
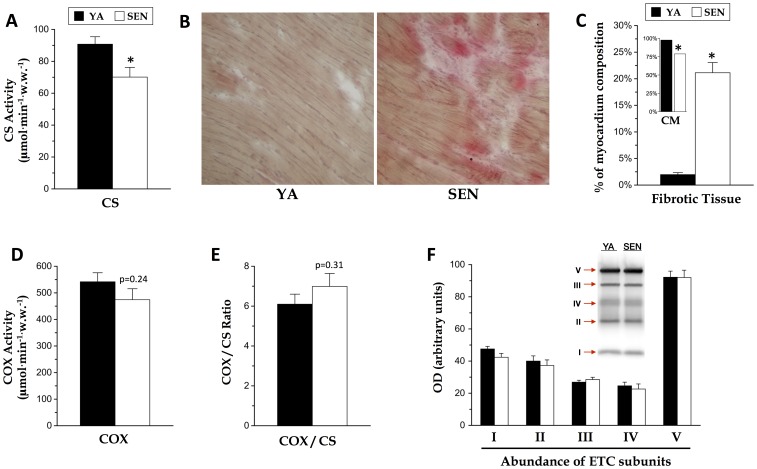
Loss of cardiomyocytes but not of mitochondrial electron transport chain content in the aging heart. (A) Citrate synthase (CS) enzymatic activity was measured on bundles of permeabilized cardiomyocytes from the left myocardium of young adult (YA) and senescent (SEN) rats. (B) Heart area occupied by fibrotic tissue (pink stain) and cardiomyocytes (brown stain) was quantified (C), with inset showing the fraction area occupied by cardiomyocytes (CM). (D) Cytochrome c oxidase (COX) enzymatic activity measured as for citrate synthase. (E) Ratio of enzymatic activities for COX and CS in YA and SEN hearts. (F) Protein abundance of electron transport chain complexes subunits was quantified by Western blots on homogenates of the left myocardium, and normalized to total soluble protein content. OD: optical density. Antibodies used were specific to complex I subunit NDUFB8 (CI), complex II subunit 30 kDa (CII), complex III subunit core 2 (CIII), complex IV subunit 1 (CIV), and complex V subunit α (CV). N = 8 animals per group, data are means ± S.E.M. * P<0.05 vs YA.

### Abundance of electron transport chain complexes

Homogenates were prepared from the left ventricle, and 5 µg of proteins was loaded into a precast 4–15% SDS-polyacrylamide gels (SDS-PAGE) (Bio-Rad, Hercules, USA). Proteins were transferred onto a membrane and incubated overnight with a premixed cocktail of polyclonal antibodies directed against representative subunits of each of the electron transport chain complexes (Mitosciences MS604, 6 μg × ml^−1^). Equal protein loading was verified using the Ponceau red stain. Membranes were washed in 0.05% Tween-PBS buffer, incubated with horseradish peroxidase-conjugated secondary antibody (dilution 1∶1000), and band densitometry was detected as previously described [24].

**Figure 2:**
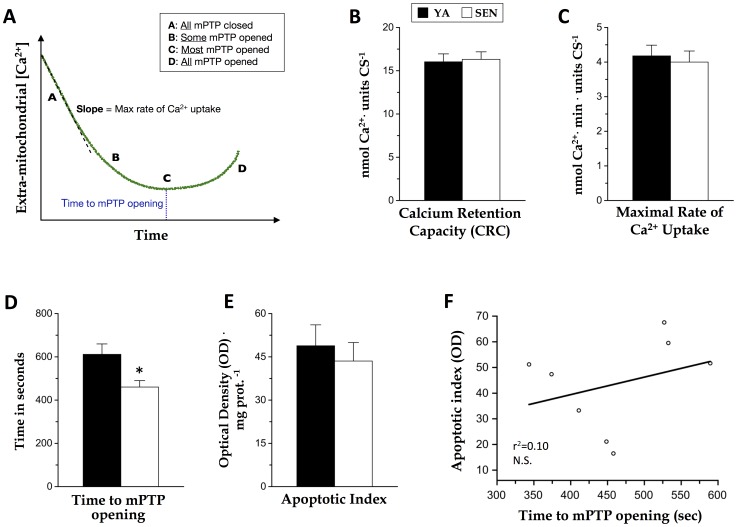
Effect of aging on mitochondrial Ca^2+^ uptake and permeability transition pore (mPTP) sensitivity in permeabilized cardiomyocytes. (A) Schematic representation of the Ca^2+^ Green fluorescence signal when a bolus of Ca^2+^ (30 µM) is applied to ghost (no contractile filaments) permeabilized cardiomyocytes; energized intact mitochondria pump Ca^2+^ enabling the measurement of Ca^2+^ uptake (decrease in fluorescence), which subsequently triggers opening of the mPTP (increase in fluorescence). (B) Ca^2+^ retention capacity assay (CRC), (C) maximal rate Ca^2+^ uptake, and (D) the time required before mPTP opening were determined from bundles of permeabilized cardiomyocytes of myocardiums from young adult (YA) and senescent (SEN) hearts. (E) Apoptotic index was determined based on DNA fragmentation quantified from homogenates of the left myocardium using an ELISA. (F) Correlation between time to mPTP opening and apoptotic index. N = 8 animals per group, data are means ± S.E.M. * P<0.05 vs YA.

### Histochemistry and quantification of fibrosis

For quantification of fibrosis, 10 μm thick cross-sections through the entire midbelly of the heart were cut using a Microme HM 550 MVP cryostat at −20°C. Sections were mounted onto glass slides and kept at −80°C until stained with Van Geison's stain, according to established methods [26]. Briefly, slides were immersed in working Weigert's iron-haematoxylin solution for 3 minutes, differentiated in tap water and immersed in Van Geison's staining solution for 2 minutes. Slides were then dehydrated and mounted to be imaged at 100× magnification on a Nikon Eclipse E400 stage (Nikon, Mississauga, ON). The anterior, lateral, and posterior portions of the subendocardium of the free wall of the left ventricle were photographed. Analysis was carried by standard stereological techniques, overlaying a 100-point grid on each image and counting which points fell on cardiomyocyte, collagen, empty space, or other. The relative proportions of cardiomyocytes versus collagen were subsequently calculated.

**Figure 3:**
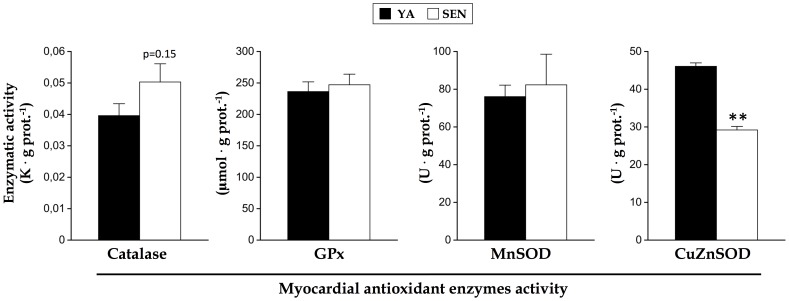
Effect of aging on activity of antioxidant enzymes in the left myocardium. (A) The activity of catalase, (B) glutathione peroxidase (GPx), (C) manganese superoxide dismutase (MnSOD or SOD2), and (D) cupper-zinc superoxide dismutase (CuZnSOD, or SOD1) were measured on homogenates of the left myocardium of young adult (YA) and senescent (SEN) hearts. N = 8 animals per group, data are means ± S.E.M. ** P<0.01 vs YA.

**Figure 4:**
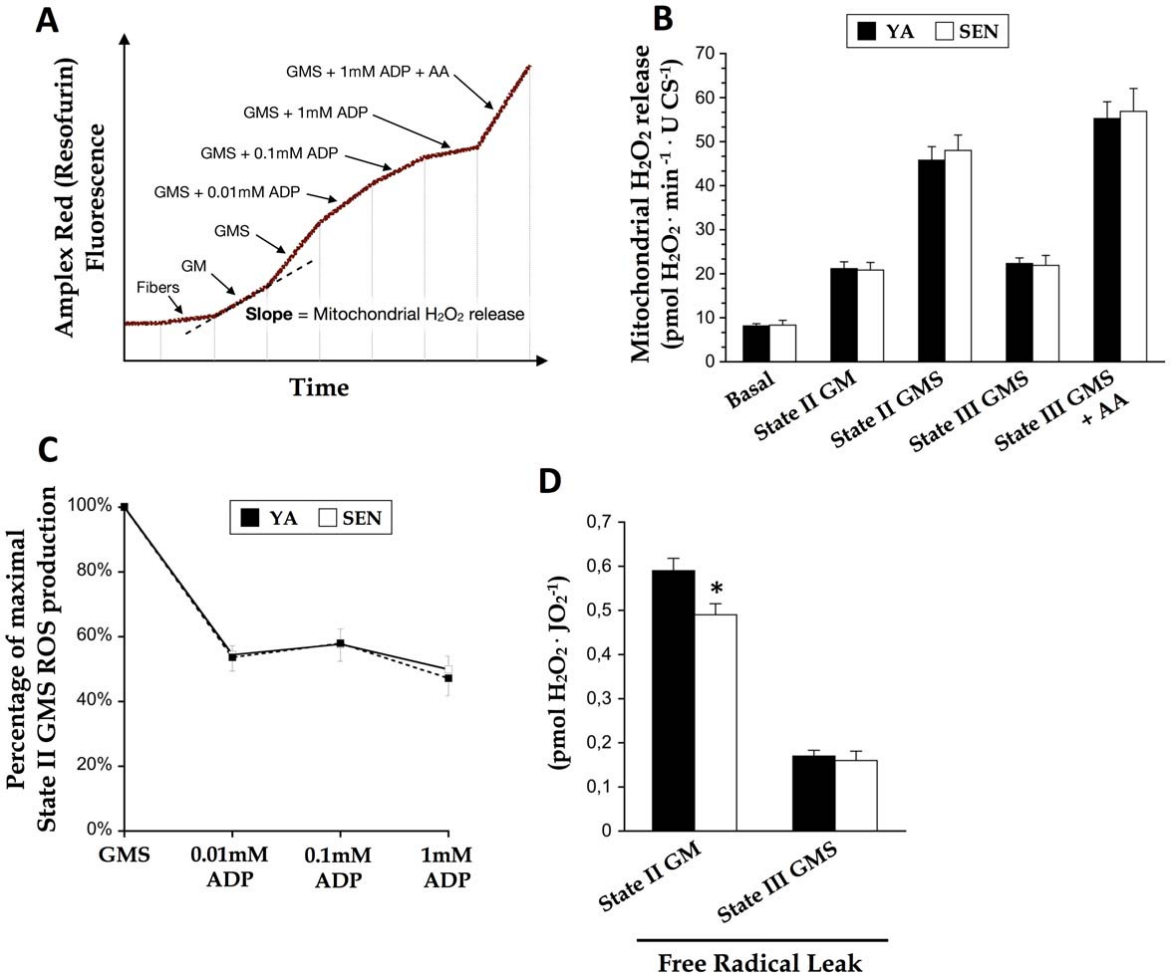
No effect of aging on mitochondrial H_2_O_2_ emission in permeabilized cardiomyocytes. (A) Schematic representation of the Amplex Red fluorescence signal and experimental substrate addition protocol on permeabilized cardiomyocytes enabling measurement of H_2_O_2_ release by intact mitochondria. (B) Mitochondrial H_2_O_2_ release in permeabilized cardiomyocytes from young adult (YA) and senescent (SEN) hearts, normalized to citrate synthase activity (CS, marker of mitochondrial content). *Basal*, endogenous production from cardiomyocytes only; *State II GM*, glutamate (10 mM) + malate (2 mM)-driven production in the absence of ADP; *State II GMS*, GM + succinate (10mM); *State III GMS*, GMS + ADP (1.11 mM); *AA*, antimycin A (10 µM). (C) Effect of incremental ADP concentrations on mitochondrial H_2_O_2_ emission. (D) Free radical leak under state II (no ADP) respiration with GM, and state III (1.11 mM ADP) respiration with GMS. N = 8 animals per group, data are means ± S.E.M. * P<0.05 vs YA.

### Citrate synthase and cytochrome c oxidase activity

Frozen myofiber bundles from respirometry assays were homogenized and used to detect spectrophotometrically the activity of citrate synthase (CS) and cytochrome c oxidase (COX) in all samples from YA and SEN animals, according to methods described previously [24].

### Preparation of permeabilized myofibers

Permeabilized myofiber bundles were prepared as previously described in [24], adapted with modifications from [27]. Briefly, half of the left ventricle was put in ice cold buffer A, the endocardium was removed and the myocardium was separated from the epicardium, with the latter portion discarded. Small bundles of cardiomyocytes from the myocardium were carefully dissected and then incubated in saponin-supplemented (0.05 mg × ml^−1^) relaxing buffer A, for 30 minutes. Permeabilized cardiomyocytes were then rinsed in stabilizing Buffer B (3×10 min) and kept on ice until use for the different measurements. All measurements of mitochondrial function were performed in duplicates, or in triplicates when the first two measures significantly diverged from one another.

**Figure 5:**
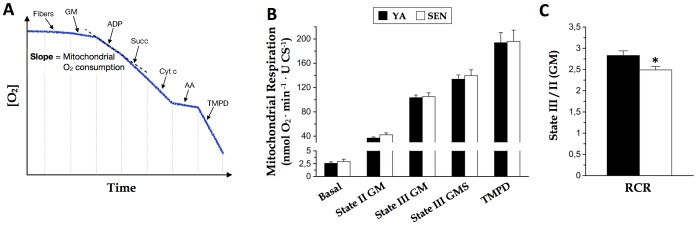
No effect of aging on mitochondrial respiration in permeabilized cardiomyocytes. (A) Schematic representation of the O_2_ concentration and experimental substrate addition protocol on permeabilized cardiomyocytes enabling measurement of O_2_ consumption by intact mitochondria. Cytochrome c was added to insure intactness of the outer mitochondrial membrane. (B) Mitochondrial respiration in permeabilized cardiomyocytes from young adult (YA) and senescent (SEN) hearts, normalized to citrate synthase activity (CS). *Basal*, endogenous respiration from cardiomyocytes only; *State II GM*, glutamate (10 mM) + malate (2 mM)-driven respiration in the absence of ADP; *State III GM*, GM + ADP (2 mM); *State III GMS*, GM + ADP + succinate (10 mM); *TMPD*, ascorbate (5 mM) + N,N,N′,N′-tetramethyl-p-phenylenediamine (0.5 mM). (C) Respiratory control ratio (RCR) determined as State III/II in GM conditions. N = 8 animals per group, data are means ± S.E.M. * P<0.05 vs YA.

### mPTP sensitivity to Ca2+

Susceptibility of mPTP opening to Ca^2+^, a known pro-apoptotic event which happens *in vivo*, was measured in permeabilized cardiomyocytes devoid of myosin (so-called “ghost” myocytes), as described previously in [24], adapted from [28], [29]. Specifically, 4–6 mg of ghost permeabilized cardiomyocytes were added to Ca^2+^ retention capacity (CRC) Buffer at 37°C, supplemented with substrates (GM) that activate the electron transport chain and allow mitochondrial-specific Ca^2+^ uptake. Measurements were performed in the presence of 0.5 mM oligomycin to inhibit mitochondrial ATP synthesis. Extramitochondrial [Ca^2+^] was monitored fluorometrically using the Ca^2+^-sensitive dye Calcium Green-5N (Molecular Probes, Invitrogen, Carlsbad, California, USA) on a spectrophotometer, and used to determine Ca^2+^ retention capacity (CRC) and time to mPTP opening. This assay was shown previously to specifically measure Ca^2+^ uptake by mitochondria and to be sensitive to the mPTP inhibitor cyclosporine A [29].

### Apoptotic index

Apoptotic index was quantified by quantifying mono- and oligo-nucleosomes in left ventricle homogenates using the cell death detection ELISA (Roche Applied Science, Germany) as per the manufacturer's instructions. Measurements were performed in triplicates, and the amount of DNA fragmentation was normalized to mg of protein determined using the Bradford assay to yield the apoptotic index.

### Mitochondrial H_2_O_2_ emission

Mitochondrial reactive oxygen species (ROS) production was estimated based on H_2_O_2_ emission from permeabilized cardiomyocytes, detected by the reaction of H_2_O_2_ with Amplex Red catalyzed by horseradish peroxidase as described in [24], and adapted from [30]. Briefly, 4–6 mg permeabilized cardiomyocyte bundles were added to Buffer Z at 37°C, to which were added 10 mM glutamate +2 mM malate (State II, GM), then 10 mM succinate +1.11 mM adenosine di-phosphate (ADP) (State III, GMS). Matching respiration values under each substrate condition were used to compute H_2_O_2_ production per O_2_ flux. H_2_O_2_ production per O_2_ flux for each muscle was determined by taking the quotient of respiration and H_2_O_2_ emission for matched substrate conditions, where the respiration and H_2_O_2_ values had each been normalized to the CS activity measured in the individual bundles used in each experiment.

### Antioxidant enzymes activities

Endogenous antioxidant enzyme activities were assessed as described previously [31]. Briefly, a portion of the remaining frozen left ventricle samples weighing 15–30 mg was homogenized at 4°C and the resulting supernatant was used in enzymatic assays of glutathione peroxidase (GPx), catalase, manganese superoxide dismutase (MnSOD, also SOD2) and cupper zinc SOD (CuZnSOD, also SOD1). To distinguish the activity of MnSOD from CuZnSOD, the SOD assay was condicted before and after incubation with NaCN.

### High-resolution respirometry

Permeabilized cardiomyocytes were used to determine mitochondrial respiratory capacity under different respiratory states, using a polarographic oxygen sensor (Oxygraph-2k, Oroboros, Innsbruck, Austria), as described in [24]. Briefly, all samples were tested in duplicates where 3.5–6 mg (wet weight) permeabilized bundles were added to the respiration chambers (one bundle in each of the two chambers) containing 2 ml of Buffer B, at 37°C. Experiments were performed at hyperoxygenated levels to prevent O_2_ diffusion limitations. A sequential substrate addition protocol was used to allow functional dissection of the electron transport system: 10 mM glutamate +2 mM malate (GM – State II), 2 mM adenosine di-phosphate (ADP – State III GM), 10 μM succinate (State III GMS), 10 μM cytochrome c, 10 μM antimycin A (AA), 5 mM ascorbate +0.5 mM N,N,N′,N′-tetramethyl-p-phenylenediamine (TMPD; an artificial electron donor to complex IV). After respiration measurements were completed, bundles were frozen in liquid N_2_ and stored at −80°C for enzymatic measurements. Respiration was expressed as picomoles•second^−1^•mg^−1^ wet weight and as picomoles•min^−1^•enzymatic unit (U)^−1^ of citrate synthase (CS) activity.

### Statistics

Between group differences were evaluated using Student's *t*-test, assuming unequal variance between groups. Statistical significance for linear regressions was evaluated using Spearman's correlation coefficient. Differences were considered significant at P<0.05. All data is reported as means ± S.E.M.

## Results

### Heart and body weights

On average, whole heart mass was 50% greater in SEN than in YA (Table 1), similar to what has been previously reported in the F344 x Brown Norway F1 hybrid rat at this age [7]. Similarly, SEN body weight was 35% greater than in YA, due to an increase in fat mass [25].

### Mitochondrial content and fibrosis

Mitochondrial content in the heart was estimated by measuring biochemical enzymatic activity of the Krebs cycle enzyme citrate synthase (CS), which was 23% lower in SEN than in YA (p<0.05) (Fig. 1A). Conversely, we detected 21.1% fibrotic tissue area in the myocardium of SEN animals, whereas fibrosis was practically absent in the YA myocardium (Fig. 1B, C). This was mirrored by an 18.9% reduction in cardiomyocyte area (Fig. 1C, inset). We take this to imply that CS activity per myocyte remained constant with aging and that the lower CS activity per wet mass in SEN was due to the myocyte replacement fibrosis. On the other hand, biochemical activity of the electron transport chain system protein cytochrome c oxidase (COX, complex IV) did not differ significantly between age groups (Fig. 1D), although it trended downward. Accordingly, COX/CS activity ratio tended to be higher (15%) in SEN heart (Fig. 1E). Assessment of electron transport chain protein abundance per mg of protein revealed no difference with age (Fig. 1F).

### mPTP sensitivity to Ca^2+^ and apoptotic index

Sensitivity of the mitochondrial permeability transition pore (mPTP) was determined by measuring calcium retention capacity (CRC) in ghost permeabilized cardiomyocytes exposed to a single Ca^2+^ challenge (Fig. 2A). When normalized per unit of CS activity (to normalize for mitochondrial content) CRC was similar between ages (Fig. 2B). Likewise, the maximal rate of mitochondrial Ca^2+^ uptake was similar between age groups (Fig. 2C). In contrast, time to mPTP opening was 25% lower in SEN than in YA (Fig. 2D), indicating greater intrinsic susceptibility to opening of the mPTP with aging. Despite the increase in mPTP sensitivity with aging indicated by the reduced time to mPTP opening, there was no detectable change in a marker of apoptosis using an ELISA to detect DNA fragmentation (mono- and oligo-nucleosomes) with aging (Fig. 2E), and there was no significant correlation between apoptotic index and time to mPTP opening (Fig. 2F).

### Antioxidant activities and mitochondrial H_2_O_2_ emission

Relative to YA, there was a trend (P = 0.12) to an increased activity of calatase in SEN heart (27%; Fig. 3A). There were no changes in either GPx (Fig. 3B) or MnSOD (Fig. 3C) with aging, but conversely, CuZnSOD activity was 37% lower in the SEN heart (Fig. 3D).

YA and SEN mitochondria in permeabilized cardiomyocytes emitted the same amount of H_2_O_2_, regardless of the substrate or inhibitor conditions used to energize mitochondria (Fig. 4A, 4B). Similarly, the reduction in H_2_O_2_ emission in response to stepwise increase in ADP-stimulated respiration was identical between age groups (Fig. 4C). Finally, when expressed per O_2_ flux to provide an index of free radical leak, mitochondrial H_2_O_2_ emission for a given O_2_ flux was lower in SEN under state II respiratory conditions (no ADP), but not different under state III (with ADP) respiration (Fig. 4D).

### Mitochondrial respiration

Mitochondrial O_2_ consumption (Fig. 5A) per mg of cardiomyocyte bundle mass was systematically ∼25% lower in SEN heart (not shown), under all respiratory conditions. Since this decline was proportional to the increase in fibrosis (and thus, reduction in cardiomyocyte fraction) with aging, this suggests no change in the respiratory capacity per cardiomyocyte with aging. When respiration was normalized per unit of CS activity, there was no difference in respiratory capacity between age groups, regardless of the respiratory state measured (Fig. 5B). However, respiratory control ratio (RCR, ration of state 3 over state 2) was lower in SEN than in YA (Fig. 5C).

## Discussion

Mitochondria have been heavily implicated in the aging of post-mitotic tissues like the heart. Indeed, changes such as the well-known cardiomyocyte replacement fibrosis occurring with aging [4], [5] have been hypothesized to be due to mitochondrial dysfunction which increases the apoptotic susceptibility of aged cardiomyocytes [16], [17], [18]. In this study, we examined multiple indices of mitochondrial function in a preparation where mitochondrial structure and intracellular interactions are preserved, and where all mitochondria are represented: saponin-permeabilized cardiomyocytes. In proportion with the loss of cardiomyocyte mass (−19%) with aging, we found a parallel decrease in CS activity per unit of heart mass, suggesting that mitochondrial content is reduced in SEN heart in direct proportion to the loss of cardiomyocytes. As such, indices of ROS emission and respiratory capacity were preserved in SEN heart after accounting for the decline in CS activity, suggesting largely maintained intrinsic mitochondrial function with aging. The only functional impairments observed in SEN cardiomyocyte mitochondria were a modestly reduced respiratory control ratio and an increased sensitivity of the mPTP (time to opening), which was not associated with more DNA fragmentation in the aged heart. Collectively, therefore, at an age where cardiomyocyte replacement fibrosis is extensive and functional impairment is becoming severe [7], our results are inconsistent with the view that dysfunctional mitochondria prevail and contribute to myocardial dysfunction in the aging heart.

### mPTP sensitivity and apoptosis

Contrary to previous data showing increased DNA fragmentation in the SEN aged F344BN rat heart [5], [17], we found no difference between age groups. This could be related to the fact that we performed the DNA fragmentation ELISA using whole ventricle homogenates, whereas Ljubicic et al. [17] used isolated cytosolic fractions of the ventricle for their analyses, and Kakarla et al. [5] used *in situ* labeling of DNA fragmentation (TUNEL). Other studies examining inbred strains of rat have also observed an increase in DNA fragmentation indicative of an increase in nuclear apoptosis in the aging heart [9], [32], [33], [34]. Given our current results suggesting no increase in nuclear apoptosis in the aging heart of the more robust F344BN hybrid rat, a model with fewer age-related pathologies [35], we suggest that this discrepancy with inbred strains may be due to the greater susceptibility to age-related pathology in inbred rodents. This line of reasoning is supported by a comparative study showing a loss of cardiac mitochondrial respiratory capacity in permeabilized cardiomyocytes in the inbred Fisher 344 rat but not in the F344BN hybrid rat [23], with the latter representing the model used in our experiments.

### ROS metabolism in the senescent heart

Consistent with other studies showing no difference in antioxidant enzyme content between young adult and senescent myocardium [17], [36], we observed only a trend to an increase in catalase activity, no change in GPx or MnSOD activity, and a decrease in CuZnSOD activity with aging. In contrast, Judge et al. found the overall activity of catalase, GPx and MnSOD from the mitochondrial fractions to be significantly higher with aging in the inbred F344 rat heart [37]. Because induction of the endogenous antioxidant enzyme machinery generally occurs in response to an increase in oxidative stress [38], our results suggest that increases in oxidative stress are likely minimal in the aging F344BN heart. This view is in agreement with the lack of change in mitochondrial ROS emission under any substrate or inhibitor conditions in the aging heart seen in the current study. Indeed, normalizing ROS emission for respiration (free radical leak) revealed a small decline in ROS emission under state II conditions with aging. Other studies in rats [16], [39], [40] and in mice [41] also reported no difference in ROS production from isolated mitochondria with aging.

However, the findings that transgenic mice over-expressing mitochondrial catalase maintain superior cardiac function with aging [42], [43] suggests that mitochondrial ROS production does play a role in cardiac alterations with aging in mice. The potential discrepancy in results between mice and rats, and between the inbred F344 and hybrid F344BN rats, suggests that the role of mitochondrial ROS in cardiac aging may differ between species. Another possible interpretation is that potential differences present *in vivo* are lost following incubation of permeabilized cells (and isolated mitochondria) in idealized buffers.

### Mitochondrial respiration

The age-related increase in fibrosis that we observed is in agreement with previous reports in rat [7], [9], [33], [34] and mouse [44]. Although upregulation of extracellular matrix genes in dilated cardiomyopathy can result from a primary mitochondrial respiratory defect [45], we did not find evidence of reduced mitochondrial oxidative capacity in SEN heart. Similarly, and consistent with a prior report by Niemann et al. [33], despite a significant decrease in CS, we found no difference in COX activity, nor in the abundance of ETC proteins in the aged heart. Therefore, unless ATP synthesis or transport is selectively altered with aging, our finding that ETC function is preserved with aging suggests that cardiac function in the SEN heart is not likely to be limited by maximal oxygen consumption.

### Permeabilized myofibers vs isolated mitochondria

The majority of prior investigations have isolated mitochondria to measure their function [16], [17], [18], [37], [41], [46], [47], a technique recently shown to exaggerate mitochondrial dysfunction in aging skeletal muscle [24]. A previous study using the same rodent model at the same ages reported a 60% lower CRC measured in isolated intermyofibrillar (IMF) and no change in subsarcolemmal mitochondria (SSM) with aging [16]. In contrast, we found no difference in CRC but a 25% decline in time to pore opening measured in permeabilized cardiomyocytes, a preparation which contains both mitochondrial sub-populations. Given the greater abundance of IMF (∼80%) to SSM (∼20%) in muscle [48], and the sensitive nature of the mPTP functional assay (statistical power sufficient to detect a change in CRC of ≥14% given a sample size of 8 animals), it is highly unlikely that our experiments would have failed to detect a change in CRC even if it was confined solely to the IMF mitochondria. As such, our results suggest that the prior studies observing a reduced CRC may have been confounded by the use of isolated mitochondria, where mitochondrial yield is only a fraction of the total mitochondrial pool and the structure of mitochondria becomes fragmented relative to *in situ* or *in vivo* conditions [22].

Like other studies using isolated mitochondria [16] or permeabilized myofibers [23], we found no decrement in maximal mitochondrial respiration in Fisher344/BN rats with age. This is in contrast with other studies using isolated mitochondria, which have found lower state III respiration in isolated IMF mitochondria from senescent myocardium [37], [46], [49], but not from SSM mitochondria [16], [37], [46], [47], [49]. Based upon evidence that that altering mitochondrial morphology during isolation alters mitochondrial function in skeletal muscle [50], it is possible that the preferential respiratory deficit in IMF cardiac mitochondria seen in these former studies could result from the disruption of more complex morphological characteristics and intracellular tethers of the IMF mitochondria in the intact cardiomyocyte.

As argued above for apoptotic susceptibility, if significant respiratory impairment existed in IMF within permeabilized cardiomyocytes, the mixture of both IMF and SS mitochondria would have diluted the effect, but the relative proportion of IMF to SS mitochondria should still have allowed us to detect a potential difference, were it to exist. Thus, the fact that some studies of isolated organelles have found age-related impairments in respiratory capacity in isolated IMF but not SS mitochondria, but that studies of intact mitochondria from permeabilized cells show no respiratory dysfunction, suggest that IMF mitochondria in the senescent myocardium might be more fragile (less resistant to the isolation stress) than the SS fraction during isolation (see [22] for a discussion). IMF mitochondria in the aged heart could be more sensitive to morphology disruption either because of i) possibly more elongated/tubular mitochondrial morphology than the globular SS mitochondria [51], [52], or ii) because of greater oxidative damage to mitochondrial proteins in the intermyofibrillar compartment [37], which could negatively impact their resilience to the isolation process. However, these speculative explanations require empirical support.

### Conclusions and Perspectives

Mitochondrial-based theories remain amongst the most widely accepted in explaining the causes of aging [1], [53], [54]. The critical question is whether the relatively mild mitochondrial dysfunction that we observe in permeabilized cardiomyocytes from very aged animals, which was limited to a reduced respiratory coupling efficiency and modestly sensitized mPTP to an apoptotic challenge, contributes meaningfully to the deleterious changes seen with aging in the heart. Our results obtained under idealized buffer conditions suggest it is unlikely that mitochondrial alterations could be the cause of the profound structural and functional alterations seen in the aging heart, and that previous results may have been confounded by the use of isolated organelles to interrogate mitochondrial function. In addition to employing methods that preserve mitochondrial morphology [22], [23], future studies should also consider the effect of aging on the ongoing removal of dysfunctional mitochondria by autophagy [6], [34], [55] and regulation of this process by mitochondrial dynamics [56], [57]. Whether mitochondrial dysfunction contributes to cardiac aging remains to be well-established and we hope that our results spur critical evaluation of this hypothesis.
